# Assessment of arterial damage in vascular Ehlers-Danlos syndrome: A retrospective multicentric cohort

**DOI:** 10.3389/fcvm.2022.953894

**Published:** 2022-10-03

**Authors:** Salma Adham, Anne Legrand, Rosa-Maria Bruno, Clarisse Billon, Violaine Dalens, Pierre Boutouyrie, Jean-Michaël Mazzella, Sonia Gueguen, Michael Frank, Tristan Mirault, Xavier Jeunemaitre

**Affiliations:** ^1^CHU Montpellier, Hôpital, Saint Eloi Service de Médecine Vasculaire, Montpellier, France; ^2^AP-HP Département de Génétique et Centre de Référence des Maladies Vasculaires Rares Hôpital Européen Georges Pompidou, Paris, France; ^3^Université Paris Cité INSERM U970 Paris Cardiovascular Research Center, Paris, France; ^4^AP-HP Unité de Pharmacologie Hôpital Européen Georges Pompidou, Paris, France; ^5^Division de Médecine Interne Département de Médecine Centre Hospitalier Universitaire de Québec-Université Laval Hôpital Saint-François d’Assise, Québec, QC Canada; ^6^Sorbonne Université Inserm RaDiCo French National Program on “Rare Disease Cohorts” Hôpital Armand Trousseau, Paris, France

**Keywords:** aortic lesions, arterial damage, medium-sized artery lesions, *COL3A1* variants, vascular Ehlers-Danlos syndrome

## Abstract

**Background:**

Vascular Ehlers-Danlos syndrome (vEDS) is a rare inherited connective tissue disorder due to pathogenic variants in *COL3A1* leading to medium-size-artery (MSA) dissection, aneurysm, rupture. Aortic lesions are rarer and less investigated. The objective was to describe the distribution of MSA and aortic lesions and the type of COL3A1 variants in a multicentric cohort of 330 adult vEDS patients.

**Methods:**

At the time of the study, 87% were alive, 60.3% were index cases, and 60.0% were women. *COL3A1* variants were identified using NGS and/or Sanger sequencing and classified according to functional consequences: 80.6% leading to dominant-negative (DN) and 19.4% leading to haploinsufficiency (HI). Imaging was systematically performed during the initial workup. Carotid mechanics were assessed by echo tracking in a subgroup of patients.

**Results:**

Arterial lesions were reported in 82.4% of the patients (*N* = 272): 83.5% had MSA lesions alone, 3.3% had aortic lesions alone, and 13.2% both. DN variants were associated with a higher prevalence of arterial lesions (*P* < 0.044), especially in supra-aortic trunks and renal arteries. The prevalence of aortic lesions in HI patients with arterial lesions was higher than that in patients with DN (*P* 0.027), but not anymore when adjusted for age (*P* < 0.559). Carotid Young’s modulus was lower in patients with DN, in association with the higher incidence of MSA lesions in this group.

**Conclusion:**

The prevalence of aortic lesions is not influenced by the *COL3A1* genotype when adjusted for age. Patients with DN variant vEDS have a higher frequency of MSA lesions, especially in supra-aortic trunks associated with lower carotid stiffness. These results support optimized care and follow-up for these vulnerable patients.

## Introduction

The Ehlers-Danlos syndromes (EDS) are a group of 13 heritable disorders characterized by three common features: tissue fragility, skin hyperextensibility, and joint hypermobility (1). Vascular Ehlers-Danlos syndrome (vEDS, OMIM #130050), one of the EDS subtypes, is a rare and severe inherited connective tissue disorder (CTD) with a prevalence estimated at 1/50 000. It accounts for approximately 5% of all EDS cases (2). Autosomal dominant transmitted pathogenic variants in the *COL3A1* gene (OMIM #120180), which encodes the proα1 (III) chain of type III collagen, leading to connective tissue fragility characterized by life-threatening arterial complications (3, 4). Characteristic facial features, easy bruising, thin translucent skin, and acrogeria might hint clinicians to search for the disease in seemingly healthy young adults with a history of spontaneous arterial dissection, aneurysm, or rupture (2). Patients with vEDS can also experience spontaneous colonic perforation or uterine rupture as their first major complication (5, 6). The type of *COL3A1* variants is associated with the phenotype in patients with vEDS. On average, dominant-negative variants (glycine missense and splice-site variants, DN) are characterized by a premature onset and a more severe course of the disease in comparison with null variants (haploinsufficiency variants, HI) (7, 8). Thus, the location and severity of arterial lesions according to genotype are essential to consider as possibly associated with this differential prognosis.

At variance with marfanoid syndromes, which are associated with aortic lesions, patients with vEDS mostly have medium-size artery (MSA) lesions (3, 9). A genotype-specific association with aortic lesions has been suggested in two vEDS case series, with HI variants more tightly associated with aortic involvement (7, 10). However, given the different ages at disease onset, it is unknown whether this association is a real difference between genetic variants or if it is facilitated by tissue degeneration and/or atherosclerotic processes associated with normal vascular aging. It is now possible to accurately and non-invasively measure the vascular aging process in large arteries such as the aorta and the carotid artery by tonometry and echo tracking (11). Our group demonstrated that, in patients with vEDS, the abnormally low intima-media thickness (IMT) and stiffness induce higher wall stress in the carotid artery (a musculo-elastic artery), thus possibly increasing the risk of arterial dissection and rupture, whereas aortic mechanic properties are not modified by the disease (12, 13). Prognosis-modifying treatments such as celiprolol can reduce carotid elasticity (14).

We report in this article the prevalence and type of arterial lesions in a large cohort of molecularly proven patients with vEDS for whom a systematic multisite evaluation (history and/or angiogram) was available. Our goal was to investigate possible differences in MSA and aortic involvement between the two main types of *COL3A1* variants (DN vs. HI) and their possible dependency on normal vascular aging (1) by providing age-adjusted results and (2) by exploring arterial mechanical properties using carotid tonometry and echo tracking in a subgroup of patients.

## Materials and methods

### Study population

RaDiCo SEDVasc is a French nationwide prospective cohort of molecularly proven vEDS set up by the Rare Disease Cohorts INSERM program on 1 December 2016 and funded by the Plan d’Investissements d’Avenir (PIA) by the Agence Nationale pour la Recherche (ANR-lO-COHO-03-01). A genetic test is performed after the patient’s written informed consent, in compliance with the French legislation on genetic diagnostic testing (French bioethics law no. 2004_80). All patients have a *COL3A1* gene mutation and are categorized according to the presence of a glycine missense or splice-site variant (dominant-negative effect pathogenic variants) or a null-variant (haploinsufficiency variants) (7, 8). Their characteristics are documented in the RaDiCo database, including medical history at the time of inclusion and prospective elements such as incident arterial lesions. The INSERM institutional review board (IRB 15–250) gave ethical clearance for conducting this study on medical records. Human subjects were involved in this retrospective cohort study, and the investigation conforms to the principles outlined in the Declaration of Helsinki.

As of 5 August 2019, 372 patients have been registered with RaDiCo SEDVasc. For the present analysis, we included patients fulfilling the following criteria:

- Age ≥ 18 years.
- Arterial questionnaire (history of arterial events) fulfilled.

### Arterial lesion diagnosis

Arterial lesions were defined as any arterial event involving MSA and/or aorta, symptomatic or not, and diagnosed by imaging either during regular follow-up or in case of an acute arterial event (15). We included both events reported in medical history at inclusion and those occurring during follow-up. The assessment of arterial lesions was obtained from either Doppler ultrasound (DUS), computed-tomography angiogram (angio-CT), and/or magnetic resonance angiography (MRA) reports.

### Arterial stiffness assessment

An arterial stiffness assessment was available for 133 patients, followed at the Hôpital Européen Georges-Pompidou, AP-HP, Paris, allowing a more in-depth arterial phenotyping based on echo tracking and tonometry, as previously described (12, 14). Briefly, regional aortic stiffness was measured by carotid-femoral pulse wave velocity (PWV) (Sphygmocor^®^, Atcor, Sydney, Australia) (16, 17).

Local carotid stiffness parameters, diameter, and IMT were obtained *via* echo tracking (18, 19). The cross-sectional distensibility coefficient (DC), carotid stiffness, and Young’s elastic modulus (Einc) were obtained using classical formulas (12). The right and left values were averaged. To investigate the presence of accelerated vascular aging in patients with vEDS, we computed predicted carotid IMT, diameter and distensibility, and carotid-femoral PWV based on age, sex, and blood pressure (BP), based on European normal reference values (20–22), and compared them to the observed ones.

### Genetic analysis

*COL3A1* molecular diagnosis was performed by Sanger sequencing (2001–2015) and then by using next-generation sequencing (NGS) (2015–2019). When skin fibroblasts were available, the deleterious effect of splice-site variants was confirmed at the mRNA level for 30 families.

The variants were classified according to their consequences at the RNA/protein level. Glycine substitutions, splice-site variants leading to in-frame exon skipping, and in-frame insertion/deletion/indels localized in the triple helix domain were classified as dominant negative (DN) variants. All variants leading to haploinsufficiency (non-sense, frameshift, and splice-site variants leading to out-of-frame exon skipping) were classified as HI variants. Details of all variants are indicated in Supplementary Table 1.

### Statistical analysis

Data were analyzed using RStudio 2017 software (RStudio, Inc.). Data are presented as numbers and percentages for qualitative variables and median and inter-quartile range (IQR) for continuous variables.

For time-independent qualitative variables, we used logistic regression with adjustment for age at first arterial lesion diagnosis. Quantitative variables were studied using ANOVA to compare the two types of *COL3A1* gene variants.

Arterial stiffness variables were compared between groups (genetic variant, presence/absence of aortic, MSA, or cerebral lesions) by ANCOVA, adjusted for age first and then for age, sex, and mean blood pressure. All *P*-values were two-sided and considered significant if the value was < 0.05. The results are presented according to the STROBE guidelines for reporting observational clinical studies (23).

## Results

### Characteristics of the patients

**FIGURE 1:**
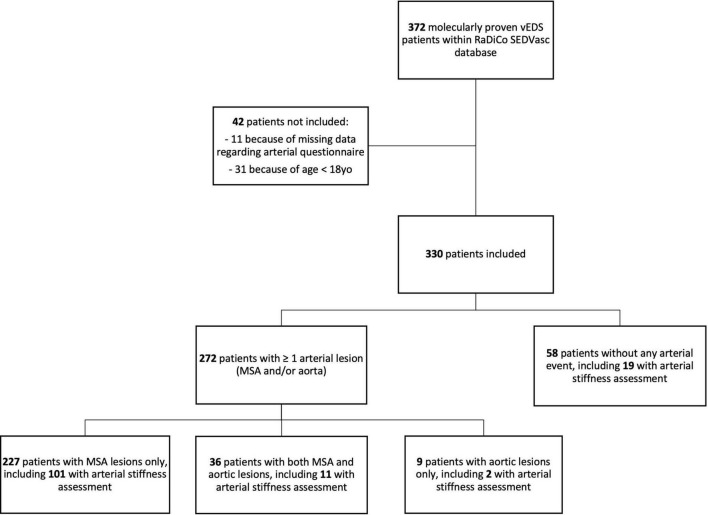
Flowchart.

Of 372 patients with vEDS in RaDiCo SEDVasc, 330 from 208 families were included, and 42 were not included because of missing data regarding arterial lesions (*N* = 11, 3.0%) or because of age < 18 years (*N* = 31, 8.3%) (Figure 1).

Two hundred and sixty-six patients (80.6%) had a DN variant in the triple helix domain (196 with a Glycine missense, 70 with a splice-site variant) and 64 (19.4%) had an HI variant (Table 1). The 45 distinct splice-site variants and in-frame insertion/deletion/indels localized in the triple helix domain were carried by 58 (82.9%) index cases. Twenty-nine occurred on the donor splice site, 14 on the acceptor splice site (2 deletions affecting both the acceptor site and the following exon), and one in the last nucleotide of the exon, disrupting the donor site (synonymous variant). The 20 distinct HI variants, among which five splice-site variants were shown to lead to HI (4 on the donor site and one on the acceptor site), were carried by 24 (37.5%) index cases. The median age at molecular diagnosis was higher in HI compared with patients with DN (*P* < 0.001). There was a low prevalence of cardiovascular risk factors at the time of molecular diagnosis (smoking, overweight, hypertension, dyslipidemia, and diabetes mellitus), similar between the two groups of variants (Supplementary Table 2). The 43 deaths reported at the time of our retrospective study all occurred in patients with a history of arterial lesions, with a significantly younger age at death in patients with DN (*P* < 0.006). Regarding the first vEDS-related event, most patients (67.9%, *N* = 224) had an arterial event without a significant difference between patients with DN and HI (*P* < 0.551). Patients with DN were younger at the first arterial onset of their disease (*P* < 0.001), but this did not lead to an earlier diagnosis of the disease compared to HI patients with the same arterial presentation at first onset (*P* < 0.128). Digestive events as the first occurrence of the disease were more frequent in patients with DN (17.7%, none in HI patients, *P* < 0.001). The median age of any first event related to vEDS (vascular event, digestive event, uterine rupture, or pneumothorax) was significantly lower in patients with DN (*P* < 0.001). The median time to molecular diagnosis after the first event did not significantly differ between genotype groups (*P* < 0.053).

**TABLE 1:** Demographic and arterial characteristics of *N* = 330 vEDS adult patients at study according to the type of *COL3A1* variants.

Characteristics^a,b^	All *N* = 330 *n* = *272 with arterial lesions* *n* = *45 with aortic lesions*	Dominant negative *N* = 266 *n* = *225 with arterial lesions* *n* = *32 with aortic lesions*	Haploinsufficiency *N* = 64 *n* = *47 with arterial lesions* *n* = *13 with aortic lesions*	*P* ^c^
**Females** *Females with arterial lesions* *Females with aortic lesions*	198 (60.0%) *157 (57.7%)* *21 (46.7%)*	156 (58.6%) *129 (57.3%)* *14 (43.8%)*	42 (65.6%) *28 (59.6%)* *7 (53.8%)*	0.307 *0.777* *0.539*
**Index cases** *Index cases with arterial lesions* *Index cases with aortic lesions*	199 (60.3%) *185 (68.0%)* *30 (66.7%)*	175 (65.8%) *161 (71.6%)* *24 (75.0%)*	24 (37.5%) *24 (51.1%)* *6 (46.2%)*	**5.5e-5** ***0.007*** ***0.069***
**Age at molecular diagnosis (years)** *Age at molecular diagnosis with arterial lesions* *Age at molecular diagnosis with aortic lesions*	36.0 (24.3–46.8) *38.0 (28.0*–*49.0)* *49.0 (35.0*–*61.0)*	34.0 (23.0–44.0) *35.0 (26.0*–*45.0)* *43.0 (31.0*–*58.0)*	47.0 (35.5–57.3) *51.0 (43.0*–*58.5)* *56.0 (51.0*–*64.0)*	**3.8e**–**7** ***1.6e***–***10*** ***0.014***
**Deceased patients** (all had arterial lesions) Age at death (years) The time between molecular diagnosis and death (years)	43 (13.0%) 38.0 (29.0–49.5) 3.0 (0.5–5)	40 (15.0%) 38.0 (28.8–42.8) 3.5 (1.0–5.3)	3 (4.7%) 53.0 (51.5–61.5) (0.0–0.5)	**0.038** **0.006** 0.089
**Arterial lesions**	272 (82.4%)	225 (84.6%)	47 (73.4%)	**0.044** ^d^
**Age at first arterial lesion (years)**	35.0 (28.0–43.0)	33.0 (27.0–40.0)	46.0 (41.0–56.0)	**1.3e**–**11**
**MSA lesions** Limb arteries Supra-aortic trunks Renal arteries Digestive arteries Coronary arteries	263 (79.7%) 140 (42.4%) 149 (45.2%) 109 (33.0%) 135 (40.9%) 11 (3.3%)	222 (83.5%) 119 (44.7%) 132 (49.6%) 100 (37.6%) 116 (43.6%) 8 (3.0%)	41 (64.1%) 21 (32.8%) 17 (26.6%) 9 (14.1%) 19 (29.7%) 3 (4.7%)	**0.044**^e^ 0.058^f^ **0.018^g^** **0.0003^h^** 0.416^i^ 0.867^j^
**Aortic lesions** Localization of aortic lesion Thorax Abdomen Type of aortic lesion Aneurysm Dissection Rupture	45 (13.6%) 21 (6.4%) 24 (7.3%) 18 (5.5%) 25 (7.6%) 2 (0.6%)	32 (12.0%) 15 (5.6%) 17 (6.4%) 12 (4.5%) 18 (6.8%) 2 (0.8%)	13 (20.3%) 6 (9.4%) 7 (10.9%) 6 (9.4%) 7 (10.9%) 0 (0%)	0.769^k^ 0.817 0.560

^a^Categorical data are presented as numbers (%).

^b^Continuous data are presented as median (interquartile range).

^c^The *P*-value for continuous variables was calculated using ANOVA for comparison between the three variants, then post-doc analysis for two-by-two comparisons using multiple comparisons of means with Tukey contrasts. The *P*-value for categorical data was calculated through logistic regression.

^d^Adjusted age groups revealed a significant difference for all vascular lesions in DN patients compared to HI patients: OR = 0.95; IQR = (0.89–1.01); *P* = 0.114.

^e^Adjusted age groups revealed a significant difference for MSA lesions in DN patients compared to HI patients: OR = 0.95; IQR = (0.89–1.01); *P* = 0.114.

^f^Adjusted age groups did not reveal a significant difference for limb artery lesions in DN patients in comparison with HI patients: OR = 1.03; IQR = (1.01–1.06); *P* = 0.004.

^g^Adjusted age groups revealed a significant difference for supra-aortic trunks lesions in DN patients in comparison with HI patients: OR = 1.00; IQR = (0.98–1.02); *P* = 0.927.

^h^Adjusted age groups revealed a significant difference for renal artery lesions in DN patients in comparison with HI patients: OR = 1.03; IQR = (1.01–1.05); *P* = 0.011.

^i^Adjusted age groups did not reveal a significant difference for digestive artery lesions in DN patients in comparison with HI patients: OR = 0.99; IQR = (0.97–1.01); *P* = 0.551.

^j^Adjusted age groups did not reveal a significant difference for coronary artery lesions in DN patients compared to HI patients: OR = 1.04; IQR = (0.99–1.09); *P* = 0.081.

^k^Adjusted age groups did not reveal a significant difference for aorta lesions in DN patients in comparison with HI patients: OR = 1.05; IQR = (1.03–1.08); *P* = 0.0002, meaning the slight difference of proportions is due to the older age of HI patients.

Characteristics were studied in the subset of adult relatives with arterial history and compared between patients with haploinsufficiency and those with dominant-negative variants. No significant difference was evidenced regarding the major characteristics (data and results not presented).

Bold values indicate significant results.

Of 330 patients in the study, 272 had arterial lesions: *N* = 263/330 (79.7%) with MSA lesions and *N* = 45/330 (13.6%) with aortic lesions (227 patients with MSA lesions only, 36 patients with both MSA and aortic lesions, and nine patients with aortic lesions only). The median age at first arterial lesion was 35.0 years [IQR, 28.0–43.0], with an earlier onset of the disease in DN compared to HI (*P* < 0.001).

The age-adjusted analysis demonstrated a higher prevalence of MSA lesions in DN (*P* < 0.044). Details of MSA lesions can be found in Table 1. There were more renal artery and supra-aortic trunk lesions in patients with DN than in patients with HI (*P* < 0.0003 and *P* < 0.018, respectively, after adjustment for age at first arterial lesion). The seemingly higher proportion of aortic lesions in HI (13/47 vs. 32/225 DN patients, *P* < 0.027) in the subgroup of patients with arterial lesions disappeared after age adjustment (*P* < 0.769, OR for age 1.05 with IQR [1.03–1.08] and *P* < 0.0001). Aortic lesions were described as 4.4% ruptures, 55.6% dissections, and 40.0% aneurysms, with as many thoracic aorta lesions as abdominal aorta lesions. No association was evidenced between the type or localization of an aortic lesion and the type of *COL3A1* variants (*P* < 0.560 and *P* < 0.817, respectively).

We compared the types of arterial lesions (*N* = 227 patients with MSA lesions without aortic lesions vs. *N* = 45 patients with aortic lesions associated or not with MSA lesions, Supplementary Table 3). Patients with aortic lesions were older at the time of molecular diagnosis and first arterial lesion (*P* < 0.001 for both, Supplementary Table 3). Death was more frequent in these patients who were also older at death (*P* < 0.032 and *P* < 0.027, respectively). Most patients with aortic lesions had MSA lesions associated (*N* = 36/45, 80.0%) without any preferential localization of the MSA lesions. A total of 451 lesions in 272 patients were identified, mainly dissections or ruptures (61.9%). Symptomatic lesions were found with similar prevalences in both variant groups (62.2 and 60% in patients with DN and HI, respectively, *P* < 0.749).

A logistic-regression model was built to explain the occurrence of aortic lesions in patients with vEDS. Men were overrepresented though not significantly [OR 1.88 with a 95% CI (0.96–3.75) and *P* = 0.067] as well as patients with older age at first arterial lesion [log10 transformation, OR 1.06 with a 95% CI (1.03–1.10) and *P* < 0.001], while the type of *COL3A1* variants was not significantly associated (Supplementary Figure 1A). Conversely, regarding the occurrence of supra-aortic trunk lesions, women [OR 1.92 with a 95% CI (1.17–3.17) and *P* < 0.010] and patients with DN [OR 2.42 with a 95% CI (1.18–5.08) and *P* < 0.017] were more at risk (Supplementary Figure 1B).

### Arterial stiffness

A subset of patients with vEDS (*N* = 133, 87.9% DN patients) underwent more detailed arterial phenotyping by tonometry and echo tracking. This group had substantially similar clinical characteristics compared to those without tonometry and echoed tracking data, except for a lower prevalence of HI variants (Supplementary Table 4).

Median aortic stiffness measured by carotid-femoral PWV was 2.0 m/s [IQR (1.2; 2.8)], higher than expected based on normal reference values for age and blood pressure. Median carotid IMT and distensibility were lower than expected [102 μm, IQR (−139; −61) and −7.2 kPa^–1^, IQR (−12.2; −0.6)], based on normal reference values, with no difference between *COL3A1* variants.

Age-adjusted aortic stiffness was not significantly different when comparing aortic and carotid mechanical properties between patients with DN and HI. Conversely, the carotid incremental elastic modulus was significantly lower, corresponding to more elastic carotid wall material in DN patients than in patients with HI. The difference remained significant even after age, sex, and mean BP adjustments (*P* < 0.014, Table 2).

**TABLE 2:** Vascular function characteristics in *N* = 133 vEDS patients according to the type of *COL3A1* variants.

Characteristics^a^	All *N* = 133	Dominant negative *N* = 117	Haploinsufficiency *N* = 16	*P* ANCOVA^b^ (age)	*P* ANCOVA^c^ (age, sex, and MBP)
**Carotid-femoral PWV (m/s)**	9.1 (7.9; 10.0)	9.0 (7.8; 10.0)	9.4 (8.7; 10.3)	0.284	0.696
**Carotid diameter (μ m)**	6,442 (5,951; 6,890)	6,453 (5,951; 6,849)	6,570 (6,136; 7,060)	0.537	0.432
**Carotid IMT (mm)**	402 (348; 474)	398 (342; 465)	449 (396; 494)	0.561	0.843
**Carotid PP (mmHg)**	43.0 (36.8; 51.0)	43.0 (37.8; 52.3)	41.5 (33.5; 51.0)	0.203	0.391
**Carotid distensibility (kPa^–1^)**	24.1 (18.0; 33.6)	25.3 (19.0; 34.5)	17.6 (14.5; 25.1)	0.343	0.246
**Carotid elastic modulus (kPa)**	**0.57 (0.43; 0.77)**	**0.56 (0.43; 0.73)**	**0.72 (0.56; 1.01)**	**0.047**	**0.014**
**Static CWS (kPa)**	88.9 (76.8; 99.7)	89.9 (77.7; 101.4)	81.2 (73.5; 90.1)	0.172	0.308
**Pulsatile CWS (kPa)**	58.0 (48.2; 70.9)	59.0 (49.7; 72.0)	52.5 (45.4; 59.9)	0.215	0.593

^a^Continuous data are presented as median (interquartile range).

^b^Adjusted age groups.

^c^Groups adjusted on age, sex, and mean blood pressure (MBP). Bold values indicate significant results.

We compared arterial stiffness parameters between vEDS patients with or without aortic lesions. Those with aortic lesions showed higher carotid-femoral PWV [10.0 m/s, IQR (8.9; 11.9) vs. 9.0 m/s, IQR (7.8; 9.8), *P* < 0.011], higher IMT [484 μm, IQR (389; 670) vs. 399 μm, IQR (348; 457), *P* < 0.002], and higher incremental elastic modulus [0.72 kPa, IQR (0.54; 0.96), vs. 0.56 kPa, IQR (0.43; 0.76), *P* < 0.006]. However, all these differences disappeared upon adjustment for age (*P* < 0.730, *P* < 0.465, and *P* < 0.378, respectively).

We then compared arterial stiffness parameters between vEDS patients with or without MSA lesions. Conversely, age-adjusted carotid-femoral PWV was significantly increased [9.3 m/s, IQR (8.4; 10.5) vs. 8.2 m/s, IQR (7.1; 9.4), *P* 0.026] and carotid distensibility significantly reduced [23.6 kPa^–1^, IQR (17.9; 32.3) vs. 28.9 kPa^–1^, IQR (23.3; 48.1), *P* < 0.008] in vEDS patients with MSA lesions; the difference in carotid distensibility persisted after further adjustment on sex and mean BP (*P* 0.012). Interestingly, when vEDS patients with supra-aortic trunk lesions were compared to those without, they showed a significantly reduced carotid diameter [6,300 μm, IQR (5,910; 6,738) vs. 6,595 μm, IQR (6,196; 6,915), *P* < 0.003 age-adjusted; *P* < 0.016 age, sex, and mean BP-adjusted].

## Discussion

Arterial aneurysm and/or rupture is the leading cause of morbimortality in vEDS, whereas medium-sized artery lesions, especially cervical and renal, are more frequent in patients with *COL3A1* DN variants, showing that aortic lesions in vEDS are mainly associated with age, possibly caused by a synergistic effect of disease-specific vascular characteristics and normal vascular aging. This hypothesis is supported by the following evidence: (1) in the overall vEDS population, the difference in the prevalence of aortic involvement in HI vs. DN patients is no longer significant when adjusted for age is performed, and (2) subgroup analysis showed that increased aortic stiffness in patients with aortic involvement is no longer significant after adjustment for age.

### Arterial lesions in vascular Ehlers-Danlos syndrome

vEDS is associated with an increased risk of devastating arterial events. As many as 82.4% of our adult patients had a record of overall arterial lesions (MSA and/or aorta) before or during follow-up since their molecular diagnosis. As expected from previous publications, patients with DN had their first arterial event at a younger age than HI patients (7, 10, and 24), but this finding did not seem to influence the time to vEDS molecular diagnosis in either group.

We also confirmed the significant difference regarding overall arterial lesions in the different groups of *COL3A1* variants (84.6% of DN and 73.4% of HI variants, respectively, *P* < 0.044, Table 1) with preferential locations in supra-aortic trunks and renal arteries in DN patients.

However, patients with HI in our cohort had a higher prevalence of arterial lesions than previously reported, a likely consequence of imaging follow-up performed systematically in symptomatic and asymptomatic patients in our referral centers. Interestingly, the same prevalence of dissections and ruptures vs. aneurysms was found in both variant groups. This finding supports the interest in a systematic imaging follow-up in vEDS patients, regardless of their genotype.

As far as mechanical arterial properties are concerned, this study confirms that vEDS is characterized by reduced carotid IMT (roughly 20%) compared to what is observed at the same age and sex in the general population. Interestingly, this reduced carotid thickness is similar between the two types of *COL3A1* variants and is thus not associated with the disease severity.

Going further into detail, patients with DN had a higher prevalence of supra-aortic trunk lesions than HI patients and had inappropriately elastic carotid arteries. In particular, Young’s elastic modulus of the carotid (i.e., the intrinsic stiffness of the wall) was lower in patients with DN variants than in those with HI variants. This finding confirms that patients with DN have a worse prognosis and suggests that this might be related to more altered arterial mechanical properties of the MSA than HI patients. Interestingly, celiprolol selectively reduces carotid elastic modulus (14) and has significantly reduced cardiovascular events in patients with vEDS (14, 15). Patients with supra-aortic trunk lesions showed a reduced carotid diameter, even after age, sex, and mean BP adjustment. This surprising finding can result from developmental anomalies, exposing themselves to local complications (25, 26), which deserves to be explored in further mechanistic studies.

### Aortic lesions

Aortic lesions in vEDS have been reported to be more at risk in patients with HI than in patients with DN (7, 8, and10). We confirmed this higher prevalence in our cohort, but as patients with HI were older than patients with DN at the diagnosis of the first arterial lesion, we tested whether age could explain this result. Indeed, the difference disappeared after age adjustment, and aortic lesion occurrence seems dependent on age in both *COL3A1* variants (DN and HI). Moreover, patients with HI and DN showed no difference in terms of age-adjusted aortic stiffness. Vascular aging on already fragile arteries might thus explain the age-dependent high frequency of aortic lesions in vEDS. Therefore, as for patients with Marfan or Loeys-Dietz syndromes, patients with vEDS should be considered at risk for aortic events when getting older, regardless of their genotype (10, 27).

## Conclusion

Vascular EDS is a rare and severe CTD mainly due to variants in the *COL3A1* gene. Arterial events in young adults and compatible physical features often lead to early vEDS molecular testing and diagnosis in patients with DN variants, in contrast to HI variants, whose first symptomatic arterial events appear later. In the retrospective analysis of the largest national registry of patients with vEDS in Europe, we found that the seemingly increased prevalence of aortic lesions in vEDS patients with HI variants resulted from their longer life expectancy and more advanced age in the disease. We also found a higher prevalence of MSA (cervical, renal) lesions in patients with DN, associated with a lower carotid Young’s elastic modulus, indicating a higher mechanical arterial fragility; this is an argument for more aggressive care and cardiovascular prevention in these particularly vulnerable patients.

## Data availability statement

The original contributions presented in this study are included in the article/Supplementary material, further inquiries can be directed to the corresponding author.

## Ethics statement

The studies involving human participants were reviewed and approved by the INSERM institutional review board (IRB 15-250). The patients/participants provided their written informed consent to participate in this study.

## Author contributions

SA, XJ, AL, and TM: conceptualization. SA, PB, R-MB, XJ, AL, and TM: methodology. SA, CB, R-MB, and AL: validation. SA and R-MB: formal analysis: SA, R-MB, MF, AL, and J-MM: investigation. PB, XJ, and SG: resources. SG: data curation. SA, R-MB, XJ, AL, and TM: writing—original draft and visualization. SA, CB, PB, R-MB, XJ, AL, and TM: writing—review and editing. XJ and TM: supervision. SA and XJ: project administration. All authors contributed to the article and approved the submitted version.

## Supplementary material

Supplementary Figure 1 **(A)** Explanatory variables for aortic lesions. **(B)** Explanatory variables for SAT lesions.

Supplementary Table 1 Identified pathogenic variants in the 330 patients included in the study.
