# Supplementary figures and images for: Assessment of arterial damage in vascular Ehlers-Danlos syndrome: A retrospective multicentric cohort
Source: Front Cardiovasc Med. 2022 Oct 3;9:953894. doi: 10.3389/fcvm.2022.953894 (PMC9573967; doi:10.3389/fcvm.2022.953894)

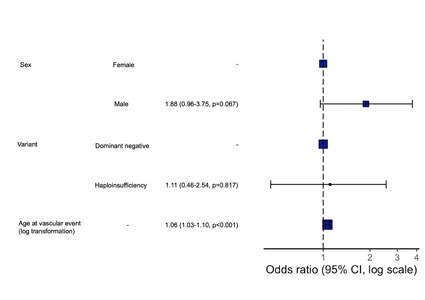

Supplement: Supplementary Figure 1 — (A) Explanatory variables for aortic lesions. (B) Explanatory variables for SAT lesions. [file Image_1.JPEG]

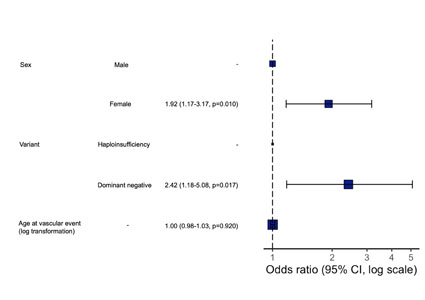

Supplement: Supplementary file 3 [file Image_2.JPEG]
