# Supplementary material for: Assessment of arterial damage in vascular Ehlers-Danlos syndrome: A retrospective multicentric cohort
Source: Front Cardiovasc Med. 2022 Oct 3;9:953894. doi: 10.3389/fcvm.2022.953894 (PMC9573967; doi:10.3389/fcvm.2022.953894)
Supplement: Supplementary file 4 [file Table_2.DOCX]

**Table S2 – Cardiovascular risk factors in N = 330 vEDS adult patients at study according to the type of *COL3A1* variant.**

| **Characteristics** ^a^ | **All**  **N=330** | **Dominant negative**  **N=266** | **Haplo-insufficiency**  **N=64** | ***P ^b^*** |
| --- | --- | --- | --- | --- |
| **Overweight (na*=110)** | 47 (14.2%)  10 (3.0%)  48 (14.5%)  34 (10.3%)  43 (13.0%) | 38 (14.3%)  9 (3.4%)  38 (14.3%)  27 (10.2%)  33 (12.4%) | 9 (14.1%)  1 (1.6%)  10 (15.6%)  7 (10.9%)  10 (15.6%) | 0.738  0.506  0.627  0.846  0.749 |
| **Diabetes (na=125)** |  |  |  |  |
| **High LDL-c (na=124)** |  |  |  |  |
| **Hypertension (na=134)** |  |  |  |  |
| **Smoking (na=111)** |  |  |  |  |

^a^ Categorical data are presented as number (%).

^b^ The *P* value for categorical data was calculated through logistic regression.

No difference in the subset of patients with arterial lesions, data not shown.

^*^ na: not available.
