# Supplementary material for: Assessment of arterial damage in vascular Ehlers-Danlos syndrome: A retrospective multicentric cohort
Source: Front Cardiovasc Med. 2022 Oct 3;9:953894. doi: 10.3389/fcvm.2022.953894 (PMC9573967; doi:10.3389/fcvm.2022.953894)
Supplement: Supplementary file 5 [file Table_3.DOCX]

**Table S3 – Characteristics and comparison of N = 227 patients with MSA lesions only (no aortic lesion) and N = 45 patients with aortic lesion (associated or not with MSA lesions).**

| **Characteristics** ^a,b^ | **Patients with arterial lesions N=272** | **No aortic lesion N=227** | **Aortic lesion N=45** | ***P*** ^c^ |
| --- | --- | --- | --- | --- |
| **Females** | 157 (57.7%) | 136 (59.9%) | 21 (46.7%) | 0.103 |
| **Index cases** | 185 (68.0%) | 155 (68.3%) | 30 (66.7%) | 0.832 |
| **Type of variant**   - Dominant negative - Haploinsufficiency | 225 (82.7%)  47 (17.3%) | 193 (86.3%)  34 (13.7%) | 32 (71.1%)  13 (28.9%) | **0.027** |
| **Age at molecular diagnosis (years)** | 38.0 (28.0-49.0) | 37.0 (27.0-46.0) | 49.0 (35.0-61.0) | **2.4e-5** |
| **Deceased**   - Age at death (years) - Time between molecular diagnosis and death (years) | 43 (15.9%)  38.0 (29.0-49.5)  3.0 (0.5-5.0) | 31 (13.7%)  38.0 (27.5-41.5)  4.0 (1.0-5.5) | 12 (26.7%)  48.5 (30.5-54.0)  2.0 (0.0-5.0) | **0.032**  **0.027**  0.221 |
| **Age at first arterial lesion (years)** | 35.0 (28.0-43.0) | 34.0 (27.0-41.8) | 43.5 (32.8-53.3) | **6.9e-6** |
| **Non-aortic arterial lesion**   - Limb arteries - Supra-aortic trunk arteries - Renal arteries - Digestive arteries - Coronary arteries | 263 (96.7%)  140 (51.7%)  149 (54.9%)  109 (40.2%)  135 (49.8%)  11 (4.1%) | 113 (50.0%)  129 (57.1%)  91 (40.3%)  117 (51.8%)  7 (3.1%) | 36 (80.0%)  27 (60.0%)  20 (44.4%)  18 (40.0%)  18 (40.0%)  4 (8.9%) | 0.441 ^d^  0.228 ^e^  0.815 ^f^  0.277 ^g^  0.240 ^h^ |

^a^ Categorical data are presented as number (%).

^b^ Continuous data are presented as median (IQR).

^c^ The *P* value for continuous variables was calculated using ANOVA. The *P* value for categorical data was calculated through logistic regression.

^d^ Adjusted age-groups did not reveal a significant difference for limb artery lesions in patients without aorta lesions in comparison with patients with aorta lesions: OR for age 1.02, IQR 1.00-1.04, *P* 0.035.

^e^ Adjusted age-groups did not reveal a significant difference for SAT lesions in patients without aorta lesions in comparison with patients with aorta lesions: OR for age 0.99, IQR 0.96-1.01, *P* 0.563.

^f^ Adjusted age-groups did not reveal a significant difference for renal artery lesions in patients without aorta lesions in comparison with patients with aorta lesions: OR for age 1.021 IQR 0.99-1.03, *P* 0.252.

^g^ Adjusted age-groups did not reveal a significant difference for digestive artery lesions in patients without aorta lesions in comparison with patients with aorta lesions: OR for age 0.99, IQR 0.97-1.01, *P* 0.502.

^h^ Adjusted age-groups did not reveal a significant difference for coronary artery lesions in patients without aorta lesions in comparison with patients with aorta lesions: OR for age 1.04, IQR 0.99-1.08, *P* 0.129.
