# Supplementary material for: Assessment of arterial damage in vascular Ehlers-Danlos syndrome: A retrospective multicentric cohort
Source: Front Cardiovasc Med. 2022 Oct 3;9:953894. doi: 10.3389/fcvm.2022.953894 (PMC9573967; doi:10.3389/fcvm.2022.953894)
Supplement: Supplementary file 6 [file Table_4.DOCX]

**Table S4 – Characteristics of the vEDS patients studied or not by carotid echotracking.**

| **Characteristics** ^a,b^ | **No echotracking data**  **N=197** | **Echotracking data**  **N=133** | ***P*** ^c^ |
| --- | --- | --- | --- |
| **Females** | 115 (58.4%) | 83 (62.4%) | 0.464 |
| **Index cases** | 116 (58.9%) | 83 (62.4%) | 0.521 |
| **Type of variant**   - Dominant negative - Haploinsufficiency | 149 (75.6%)  48 (24.4%) | 117 (87.9%)  16 (12.0%) | **0.006** |
| **Age at molecular diagnosis (years)** | 37.0 (23.0-49.0) | 35.5 (25.0-44) | 0.548 |
| **Deceased ^*^**   - Age at death (years) - Time between molecular diagnosis and death (years) | 33 (16.8%)  40.0 (29.0-52.0)  2.0 (0.0 – 5.0) | 10 (7.5%)  36.0 (28.3-38.0)  5.5 (4.3-6.8) | **0.017**  0.248  0.221 |
| **Age at first arterial lesion (years)** | 35.0 (26.0-43.0) | 33.0 (27.3-42.0) | 0.355 |
| **Arterial lesions** | 158 (80.2%) | 114 (85.7%) | 0.389 |
| **MSA lesions**   - Limb arteries - SAT - Renal arteries - Digestive arteries - Coronary arteries | 151 (76.6%)  81 (41.1%)  76 (38.6%)  58 (29.4%)  75 (38.1%)  5 (2.5%) | 112 (84.2%)  59 (44.4%)  73 (54.9%)  51 (38.3%)  60 (45.1%)  6 (4.5%) | 0.389  0.910  **0.015**  0.187  0.494  0.335 |
| **Aorta lesions** | 32 (16.2%) | 13 (9.8%) | 0.099 |

^a^ Categorical data are presented as number (%).

^b^ Continuous data are presented as median (IQR).

^c^ The *P* value for continuous variables was calculated using ANOVA. The *P* value for categorical data was calculated through logistic regression.

^*^ Deceased at the time of this retrospective study.
